# Patient knowledge, attitude and satisfaction with clear aligners versus traditional braces: A comparative study

**DOI:** 10.6026/973206300212496

**Published:** 2025-08-31

**Authors:** Shaivi Sharma, Amit Tiwari, Shivkant Mishra, Prerna Raje Batham, Himanshu Sharma, Sania Khan

**Affiliations:** 1 Department of Dentistry, Amaltas Institute of Medical Science, Bangar Village, Ujjain Dewas road Dewas, Madhya Pradesh, India; 2 Department of Orthodontics and Dentofacial Orthopaedics, Index Institute of Dental Sciences, Indore, Madhya Pradesh, India; 3 Department of Orthodontics and Dentofacial Orthopedics, Triveni Institute of Dental Sciences Hospital and Research Center, Bilaspur, Chhattisgarh, India; 4 Department of Orthodontics and Dentofacial Orthopedics, Government College of dentistry, Indore, Madhya Pradesh, India; 5 Department of Conservative Dentistry and Endodontics, Modern Dental College and Research Centre, Indore, Madhya Pradesh, India; 6 Department of Orthodontics & Dentofacial Orthopedics, Sri Aurobindo College of Dentistry, Indore, Madhya Pradesh, India

**Keywords:** Clear aligners, traditional braces, orthodontic treatment, patient satisfaction, patient knowledge, patient attitude, comparative study

## Abstract

Patient knowledge, attitude and satisfaction influence the experience of orthodontic care with clear aligners versus traditional braces. A cross-sectional survey of 200 patients revealed that those treated with clear aligners had significantly higher knowledge scores, more positive attitudes toward comfort and aesthetics and greater overall satisfaction. Satisfaction rates were 92% for clear aligners and 76% for braces, with comparable confidence in treatment efficacy between both groups. Data was analyzed using SPSS with statistical significance set at p < 0.05. Thus, we show the importance of patient-centered care and individualized treatment planning in orthodontics.

## Background:

In order to improve oral function, psychological health and dental aesthetics, orthodontic treatment is essential. Because of their demonstrated efficacy and adaptability in handling complicated orthodontic cases, traditional fixed orthodontic appliances also referred to as metal braces have been the accepted treatment option for a number of malocclusion types for many years [1]. However, the advent and quick uptake of clear aligner therapy in recent years has brought about significant changes in the orthodontic field. Compared to traditional braces, clear aligners, like the Invisalign® system, are more aesthetically pleasing and practical. Adults and teenagers looking for less obvious orthodontic solutions have come to favour these detachable, transparent trays, which are specially made to gradually move teeth [2]. Because the aligners can be taken out during meals and brushing, their primary benefits include improved appearance, improved oral hygiene and fewer dietary restrictions [3]. Despite these advantages, clear aligners may not be able to treat more complicated cases and successful results depend on high patient compliance [4]. Patient-centered care has grown in importance in contemporary orthodontic practice. Compliance, the course of treatment and the results are greatly influenced by variables like a patient's attitude towards appliances, level of satisfaction and knowledge of available treatment options [5]. Patients are more likely to actively participate in their care and adhere to instructions when they are better informed about their orthodontic treatment, which increases patient satisfaction and enhances treatment efficacy [6]. Numerous factors, including comfort, visibility, pain, ease of maintenance and social perception, affect attitudes towards orthodontic appliances. For example, research indicates that patients who wear clear aligners report higher levels of comfort and social acceptance than those who wear fixed braces [7]. Patient satisfaction, however, can be arbitrary and is contingent upon both the daily experience of the treatment process and the mechanical result. Efficiency and durability may be more important to some people than comfort and beauty. Research comparing patient-reported outcomes, such as knowledge, attitude and satisfaction, between clear aligner and traditional brace users is relatively lacking, despite the well-established clinical effectiveness of clear aligners. Although study did not assess patients' level of expectations or knowledge, it did show higher levels of satisfaction with aligners in terms of appearance and social acceptability [8]. This emphasises the necessity of assessing patient experiences in a more comprehensive manner. Therefore, it is of interest to describe compare patient satisfaction, attitudes and knowledge regarding clear aligners and traditional braces.

## Methodology:

The purpose of this cross-sectional study was to evaluate and contrast patient satisfaction, attitude and knowledge about clear aligners versus traditional braces. Using stratified random sampling, 200 participants were chosen for the study: 100 patients who received treatment with clear aligners and 100 who received traditional metal braces. Participants had to be between the ages of 16 and 40, have undergone orthodontic treatment for at least six months and give their informed consent in order to be eligible. Individuals with incomplete responses, craniofacial abnormalities, or mixed treatments were not included. A structured, self-administered questionnaire comprising four sections demographic data, orthodontic treatment knowledge, appliance attitude and satisfaction with treatment results was used to collect data over the course of three months in private orthodontic clinics and a university dental hospital. Both multiple-choice and Likert-scale questions were used to gauge responses. Orthodontic specialists validated the questionnaire and it was piloted for reliability (Cronbach's alpha = 0.82). The Institutional Review Board granted ethical approval and participant confidentiality was rigorously upheld. Descriptive statistics, chi-square tests for categorical data, t-tests for continuous variables and Mann-Whitney U tests for ordinal responses were all used in the data analysis, which was conducted using SPSS version 26.0. Statistical significance was defined as a p-value of less than 0.05.

## Results:

The study included 200 participants, with 100 patients in each group receiving either traditional braces or clear aligners. There were no statistically significant differences in age or gender distributions between the two groups (p > 0.05). Patients in the clear aligner group demonstrated significantly higher knowledge scores than those in the traditional braces group (mean score: 8.2 ± 1.1 vs. 6.9 ± 1.5; p < 0.001). They also showed greater awareness regarding treatment compliance, duration and maintenance. Attitudes assessed using a 5-point Likert scale revealed that clear aligner users reported significantly more positive perceptions, particularly in comfort (4.5 vs. 3.1; p < 0.001), aesthetics (4.8 vs. 3.2; p < 0.001) and ease of oral hygiene (4.7 vs. 3.0; p < 0.001). Although traditional braces users expressed slightly higher confidence in treatment effectiveness, the difference was not statistically significant (4.4 vs. 4.2; p = 0.08). Overall satisfaction was notably higher in the clear aligner group, with 92% reporting being "very satisfied" or "satisfied," compared to 76% in the braces group (p = 0.012). Factors contributing to higher satisfaction among aligner users included ease of cleaning, ability to eat normally and reduced discomfort. These findings are detailed in Table 1 (see PDF).

## Discussion:

The purpose of this study was to compare patient satisfaction, attitude and knowledge between those receiving traditional fixed orthodontic appliances and those receiving clear aligners. Clear aligner users typically report higher knowledge levels, more positive attitudes and greater overall satisfaction, according to the findings, which show a statistically significant difference between the two groups across all three measured domains. The study's most noteworthy finding is that people who use clear aligners have a higher knowledge score than people who wear traditional braces. This could be because patients who wear clear aligners take a more active part in their treatment, which usually entails getting thorough usage and maintenance instructions because the aligners are removable. According to earlier research, patients who actively research treatment options prior to beginning aligner therapy are typically older, better educated and frequently wealthier [9]. Furthermore, digital treatment simulations and regular progress updates are common features of clear aligner systems like Invisalign®, which may help people become more aware of and comprehend the procedure [10]. Treatment compliance and patient satisfaction are greatly impacted by attitudes regarding orthodontic appliances. Patients who received treatment with clear aligners in this study expressed more positive opinions about oral hygiene, comfort and appearance. These results are in line with those of Ziuchkovski *et al.*, who found that clear aligners were much more comfortable and aesthetically pleasing than fixed appliances [11]. Aligners' discrete appearance helps people feel less socially anxious, especially working adults and teenagers who are worried about how their peers will see them [12]. Another area where clear aligners performed better than braces was comfort. Wearing aligners is less painful, especially in the beginning of treatment; because they usually apply softer forces and don't have brackets or wires that can irritate soft tissues [13]. Patients who used clear aligners reported much greater levels of overall satisfaction. This finding is consistent with earlier research that found that clear aligner therapy is linked to higher patient satisfaction ratings because it promotes better oral hygiene habits, less dietary restrictions and increased comfort [14]. Because aligners are detachable, patients can continue their regular eating and oral hygiene routines, which are frequently interrupted by fixed appliances. Adult patients, who might be more sensitive to such limitations, should pay special attention to these lifestyle conveniences.

Additionally, patients who use aligners frequently experience a greater sense of control over their course of treatment. The impression of a contemporary, effective treatment experience is enhanced by features like digital treatment plans and shortened clinic visits. This is consistent with research by Liu *et al.*, who found that functional and psychological benefits, such as improved self-image and decreased treatment visibility, were associated with patient satisfaction in aligner therapy [15]. Patients undergoing orthodontic treatment with clear aligners reported higher satisfaction levels, citing improved aesthetics, comfort, and ease of oral hygiene compared to traditional braces. The study also highlighted better patient knowledge and a more favorable attitude toward aligners. These findings emphasize the growing preference for aligners in modern orthodontic care [16]. Clinical practice will be significantly impacted by these findings. Orthodontists should take into account the patient's expectations, preferences and lifestyle in addition to the clinical requirements of a case. Giving patients thorough information about both treatment options can aid in their decision-making, which may improve adherence and increase patient satisfaction. Although they might not be appropriate for every kind of malocclusion, a growing percentage of orthodontic patients find clear aligners to be a desirable alternative due to their psychological and lifestyle advantages. Additionally, the study's finding that knowledge and satisfaction are positively correlated emphasises how crucial patient education is. To match expectations with likely results, clinicians should take the time to explain the workings, restrictions and duties related to each type of appliance. This study has certain limitations in spite of its advantages. Because responses may be swayed by patient recall or the desire to give socially acceptable answers, using self-reported questionnaires may introduce bias. Furthermore, the study did not distinguish between various clear aligner brands or case complexity variations, which could affect how patients perceive the product. Longitudinal designs should be used in future research to assess how attitudes and satisfaction change during and after treatment.

## Conclusion:

Clear aligners were associated with higher patient knowledge, more positive attitudes and greater satisfaction compared to traditional braces. While aligners enhance the overall patient experience, treatment success still depends on proper case selection and patient compliance. Thus, we show the importance of individualized counseling and shared decision-making in orthodontic care.

## References

[1] Keim RG (2014). J Clin Orthod..

[2] Papadimitriou A (2018). Prog Orthod..

[3] Elkholy F (2020). Angle Orthod..

[4] Rossini G (2015). Angle Orthod..

[5] Bos A (2005). Eur J Orthod..

[6] Oliveira CM (2008). Community Dent Oral Epidemiol..

[7] Miller KB (2007). Am J Orthod Dentofacial Orthop..

[8] Hannah Chong (2025). Eur J Orthod..

[9] Boyd RL. (2007). J Clin Orthod..

[10] Nahoum HI. (1964). NY State Dent J..

[11] Ziuchkovski JP (2008). Am J Orthod Dentofacial Orthop..

[12] Kiyak HA. (2008). J Dent Educ..

[13] Shalish M (2012). Angle Orthod..

[14] White DW (2017). Angle Orthod..

[15] Liu J (2021). BMC Oral Health..

[16] AlMogbel A. (2025). J Pharm Bioallied Sci..

